# Neonatal Safety of Elective Family-Centered Caesarean Sections: A Cohort Study

**DOI:** 10.3389/fped.2018.00020

**Published:** 2018-02-12

**Authors:** Ilona C. Narayen, Estelle E. M. Mulder, Kim E. Boers, Jeroen J. van Vonderen, Vera E. R. A. Wolters, Liv M. Freeman, Arjan B. Te Pas

**Affiliations:** ^1^Division of Neonatology, Department of Pediatrics, Leiden University Medical Center, Leiden, Netherlands; ^2^Department of Pediatrics, Isala Clinics, Zwolle, Netherlands; ^3^Department of Obstetrics, Bronovo Hospital, The Hague, Netherlands; ^4^Department of Pediatrics, Reinier de Graaf Gasthuis, Delft, Netherlands; ^5^Department of Obstetrics, Leiden University Medical Center, Leiden, Netherlands

**Keywords:** neonatal outcome, caesarean section, neonate, newborn, family-centered practice

## Abstract

**Background:**

Although little data are available concerning safety for newborns, family-centered caesarean sections (FCS) are increasingly implemented. With FCS mothers can see the delivery of their baby, followed by direct skin-to-skin contact. We evaluated the safety for newborns born with FCS in the Leiden University Medical Center (LUMC), where FCS was implemented in June 2014 for singleton pregnancies with a gestational age (GA) ≥38 weeks and without increased risks for respiratory morbidity.

**Methods:**

The incidence of respiratory pathology, unplanned admission, and hypothermia in infants born after FCS in LUMC were retrospectively reviewed and compared with a historical cohort of standard elective cesarean sections (CS).

**Results:**

From June 2014 to November 2015, 92 FCS were performed and compared to 71 standard CS in 2013. Incidence of respiratory morbidity, hypothermia, temperatures at arrival at the department, GA, and birth weight were comparable (ns). Unplanned admission occurred more often after FCS when compared to standard CS (21 vs 7%; *p* = 0.03), probably due to peripheral oxygen saturation (SpO_2_) monitoring. There was no increase in respiratory pathology (8 vs 6%, ns). One-third of the babies were separated from their mother during or after FCS.

**Conclusion:**

Unplanned neonatal admissions after elective CS increased after implementing FCS, without an increase in respiratory morbidity or hypothermia. SpO_2_ monitoring might have a contribution. Separation from the mother occurred often.

## Introduction

During standard cesarean sections (CS), the mother is not able to witness the birth of her baby, and the first physical contact between mother and baby is postponed. In the Leiden University Medical Center (LUMC), infants were taken to the resuscitation table for evaluation directly after cord clamping in the case of elective CS, and the earliest moment for skin-to-skin contact with mother and initiation of breast feeding would be at the post-anesthetic recovery, after completion of the surgery of mother. However, studies have shown that mothers express less short-term and long-term satisfaction with the birth after CS when compared to vaginal births (1). Also, a higher chance of bonding problems and feeding difficulties was demonstrated after CS in comparison to vaginal births (1, 2). In 2008, the “natural cesarean” approach was first described to improve this (3). During this CS, the curtain is dropped at the moment of birth, so parents can see their baby being born (3). The infant is then dried and placed on mother’s chest directly and breast feeding can be initiated immediately. To many obstetricians, “natural” CS was considered a misnomer and the name of the procedure was changed into “family centered” CS.

In the following years, the family centered CS (FCS) has been implemented in many hospitals as an alternative to standard CS for low-risk elective CS. However, there are little data available whether the safety of the FCS procedure for mothers and newborns is comparable to the standard CS. Although there is a concern for an increased risk for hypothermia, studies show conflicting results (4). A recent study in a regional hospital in the Netherlands showed a decrease in admittance to the pediatric ward, and less neonatal infections were suspected after implementing FCS (5). No studies have been performed to assess other neonatal complications after FCS, including the risk of respiratory distress. It is known that infants born after an elective CS are at increased risk for respiratory pathology. Because of the position of the infant on the mother’s chest, with flexion of the neck, there were concerns for obstruction, leading to a less effective transition. Also, the infants are not stimulated and have direct skin-to-skin contact, which might decrease the stress level necessary for transition after an elective CS.

Since June 2014, FCS are performed as a pilot in the LUMC for singleton pregnancies at a gestational age (GA) of ≥38 weeks without risk factors for respiratory complications. In order to assess neonatal safety of FCS, we evaluated the incidence of hypothermia, respiratory pathology, and unplanned neonatal admissions after FCS and compared this with a historical cohort when standard CS was performed.

## Materials and Methods

### Study Design and Population

From June 2014, FCS were offered in the LUMC, a tertiary hospital, as an alternative to standard CS to women with an uncomplicated singleton pregnancy planned for elective CS at a GA of ≥38 weeks. In this retrospective cohort study, we compared the neonatal outcome of all infants born after FCS performed between July 2014 and November 2015 to our existing cohort of all singleton low-risk elective standard CS performed at a GA of ≥38 weeks performed between January 2013 and January 2014.

Multiple pregnancies and elective CS with expected complications for mother and/or child, such as maternal type 1 Diabetes mellitus or severe fetal congenital anomalies, were not eligible for FCS and were excluded from both groups.

### Outcome Measurements

Primary outcome was the safety for newborns, consisting of incidence of hypothermia, respiratory pathology, and rate of unplanned admissions at the Neonatal Intensive Care Unit. Rectal temperature below 36.5°C at arrival at the ward was defined as hypothermia. Respiratory pathology was defined as having clinical symptoms of respiratory insufficiency, including tachydyspnea or retractions. In order to make our results between the defined groups comparable, infants without clinical signs, but with only low oxygen saturation [peripheral oxygen saturation (SpO_2_)] measured during the procedure were not classified as having respiratory pathology in our analysis, since SpO_2_ was not monitored as standard practice during the procedure of standard CS, but only in the first 10 min after birth in a study cohort (6).

Secondary outcomes were as follows: median Apgar scores, temperature 10 min after birth and after arrival at the ward, need for respiratory support at birth, reasons for admission at the neonatal department and therapy needed.

### Procedure of FCS

During FCS, the ambient temperature of the operation room was increased, and the mother was covered with a forced-air warming system. The operation curtain was dropped at the moment of delivery, in order to enable the parents to see their baby. Delayed cord clamping was performed, and the baby was subsequently laid down in a sterile cradle and dried by the pediatric resident. A pulse oximetry sensor was attached at the right wrist of the newborn and direct skin-to-skin contact was provided, by laying the newborn on the mother’s bare chest. Heart rate (HR) and SpO_2_ of the newborn were obtained during the entire surgery of mother. The pediatric resident stayed 10 min after birth and longer if needed. The obstetric nurse was responsible for monitoring the infant after this period. Clinical symptoms of pathology, a HR < 100 bpm or SpO_2_ < 92% after 10 min after birth were set as cutoff value for the obstetric nurse to call the pediatric resident.

### Procedure of Standard CS

During standard CS delayed cord clamping was performed, the infant was shortly shown to the mother and consecutively taken to the resuscitation table for evaluation by a pediatric resident. After first assessment and stabilization if needed, the infant was laid in an incubator and taken to the maternity ward after, where an obstetric nurse was responsible for monitoring the infant. Pulse oximetry was performed only based on the judgment of the pediatric resident or as part of an ongoing study (6). First possibility of skin-to-skin contact with mother was after the procedure at the post-anesthetic recovery. Depending on the type of anesthesia and condition of mother and child, this usually occurs 1–2 h after birth, and feeding was initiated after reunion of mother and child.

### Data Collection

All data were retrieved from the medical charts from the newborns and/or mothers and anonymized collected in a database (IBM SPSS Statistics version 23) (IBM Software, New York, NY, USA, 2015). GA, birth weight, Apgar score, temperature after 10 min and at arrival at the department, HR and SpO_2_ 10 min after birth of infants born after FCS were compared to the standard CS group.

### Statistical Analysis

Quantitative data were presented as median (range/IQR), mean ± SD or number (percentage) where appropriate. The data were compared using Student’s *t*-test, chi-square test, or Mann–Whitney *U* test based on the distribution. Statistical analyses were performed with IBM SPSS Statistics version 23 (IBM Software, New York, NY, USA, 2015).

### Ethical Considerations

In the Netherlands, no ethical approval is required for anonymized studies with medical charts and patient data that were collected and noted for standard care. The LUMC Medical Ethics Committee provided a statement of no objection for obtaining and publishing the anonymized data.

## Results

In the study periods, 98 infants were born after FCS, but 6/98 were born at a GA between 37 and 38 weeks and therefore excluded. The infants born after FCS were compared to 71 infants born after standard CS at a GA of at least 38 weeks. The median (IQR) GA was 39^+0^ (38^+6^–39^+2^) weeks^+days^ vs 39^+0^ (38^+3^–39^+3^) weeks^+days^ (ns), and mean (SD) birth weight was 3,556 (470 g) vs 3,472 (414) g after FCS vs standard CS, respectively (ns). The median (range) Apgar scores at 1 and 5 min were 9 (6–10) and 9 (8–10) after FCS and 9 (2–10) and 10 (5–10) after standard CS (*p* = 0.43, *p* = 0.02, respectively).

Unplanned admission occurred in 20.7% (19/92) of the infants born after FCS when compared to CS 7.0% (5/71) of the infants after standard CS (*p* < 0.05) (Table 1). In the FCS group, 37% (7/19) of the unplanned admitted infants had respiratory pathology, compared to 80% (4/5) after standard CS (ns) (Table 1). The incidence of hypothermia at arrival at the ward was comparable (ns) (Table 1). The temperature 10 min after birth was not measured after standard CS, but was normal after FCS (Table 1). Also, temperature at arrival on the ward was normal and not statistically different between the groups.

**Table 1:** Outcome of infants with FCS.

	FCS (*n* = 92)	Standard CS (*n* = 71)	*p*-Value
Gestational age, weeks^+days^ (IQR)	39^+0^ (38^+6^–39^+2^)	39^+0^ (38^+3^–39^+3^)	0.45
Birth weight, g (SD)	3,556 (470)	3,472 (414)	0.24
Temp 10 min after birth, °C (SD)	36.8 (0.3)	–	–
Temp at department, °C (SD)	36.8 (0.4)	36.7 (0.6)	0.40
Hypothermia, *n* (%)	12 (13.0)	13 (18.3)	0.39
Apgar 1 (range)	9 (6–10)	9 (2–10)	0.43
Apgar 5 (range)	9 (8–10)	10 (5–10)	0.02
Taken to resuscitation table, *n* (%)	21 (22.8)	All, standard	-
Respiratory support given in OR, *n* (%)	12 (13.0)	9 (12.7)	1.0
Unplanned admission neonatology, *n* (%)	19 (20.7)	5 (7.0)	0.03
Respiratory pathology, *n* (%)	7 (7.6)	4 (5.6)	0.76
Duration of hospitalization, days (IQR)	3.0 (1.0–3.0)	2.5 (1.3–4.5)	0.76

*OR, operation room; temp, temperature; CS, caesarean section(s); FCS, family-centered CS*.

*Values are given as median (IQR or range), mean (SD) or number (%) were appropriate*.

**Figure 1:**
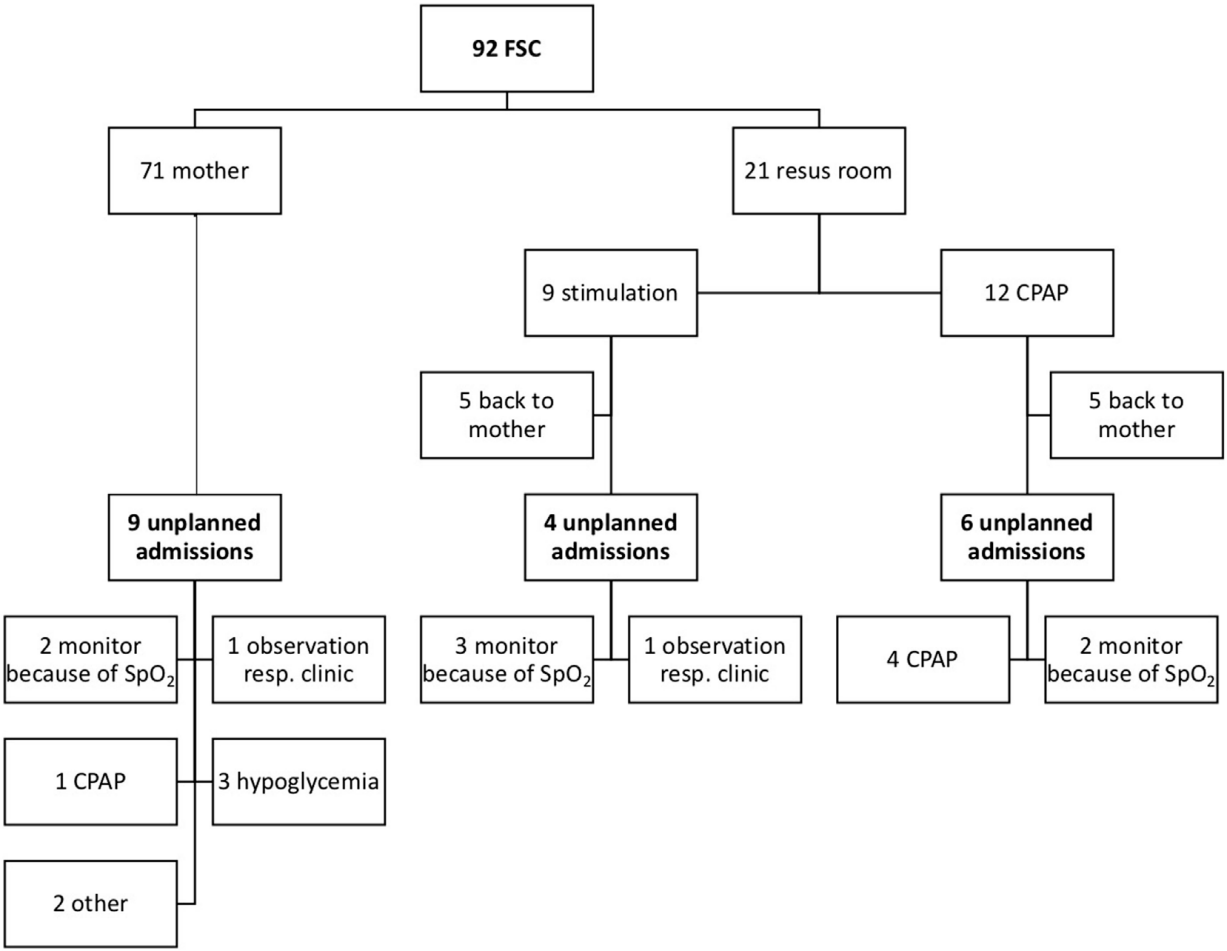
Overview of neonatal outcome after family centered-caesarean section (FCS). A flowchart of the neonatal outcome after FCS is shown. Resus room, resuscitation room; Resp clinic, clinical symptoms of respiratory pathology.

Figure 1 demonstrates the neonatal outcome of infants born after FCS. One third of the babies were separated from their mother in the delivery room and/or at the maternity ward after FCS. Monitor observation because of low SpO_2_ was the reason for admission in 7/19 newborns with unplanned admissions. Seven infants needed CPAP or had clinical symptoms of respiratory pathology. Five infants were admitted for other pathology, which could not be linked to the FCS, including three infants with hypoglycemia, one with feeding difficulties, and one with hyperbilirubinemia.

**Figure 2:**
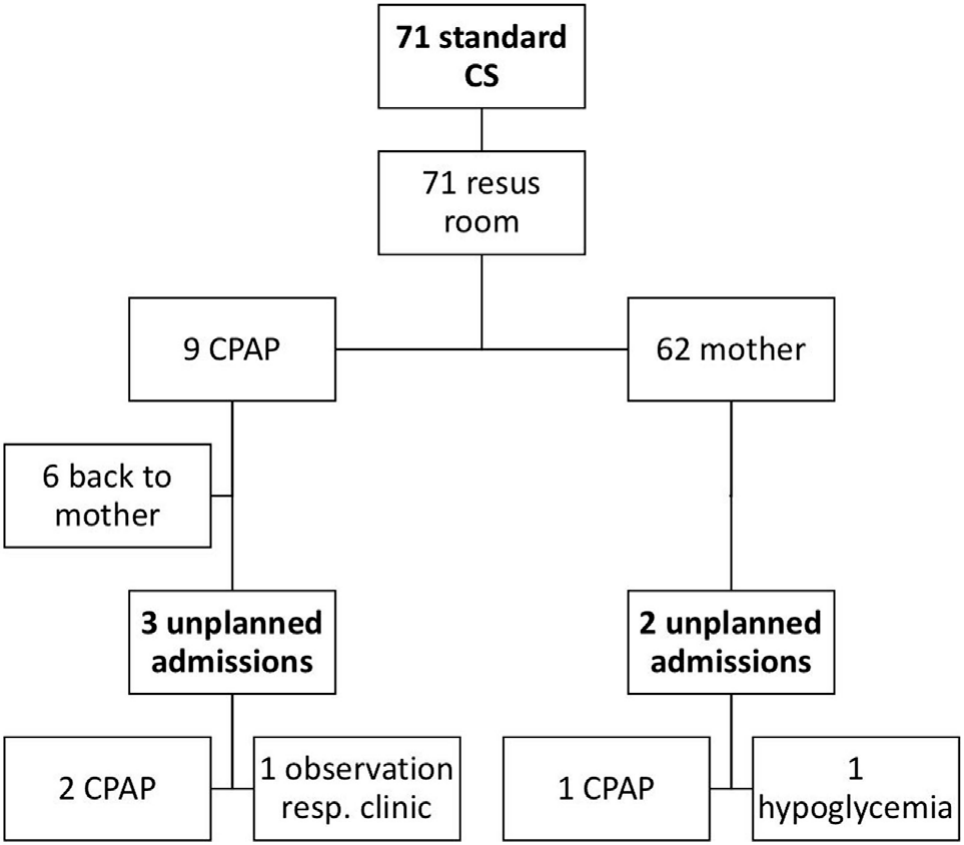
Overview of neonatal outcome after standard cesarean sections (CS). A flowchart of the neonatal outcome after standard CS is shown. Resus room, resuscitation room; Resp clinic, clinical symptoms of respiratory pathology.

Figure 2 demonstrates the neonatal outcome of infants born after standard CS; 9/71 infants needed CPAP in the delivery room. Five infants were unplanned admitted to the neonatal ward, of which three infants needed CPAP, one infant had clinical symptoms of respiratory morbidity and low SpO_2_, and one infant had hypoglycemia.

## Discussion

Implementation of FCS did not lead to an increase in respiratory pathology or hypothermia for newborns, but the rate of unplanned admission increased. Since the SpO_2_ was not monitored in the standard CS procedure, monitor observation for infants with low SpO_2_ but no other clinical symptoms has contributed to an increase in the admittance rate in the FCS group. In retrospect admittance might have been avoidable, implicating that newborns with low SpO_2_, but with a fast improvement, could stay with their mother, when there is pulse oximetry observation.

No prospective studies assessing safety of FCS have been performed. Recently, Posthuma et al. reported in a retrospective study a decrease in incidence of suspected neonatal infections and a decrease in admission to the pediatric department (5). Although these infants were not monitored during the procedure of FCS, the authors stated that “hands-off management” might enable the infant to adapt physiologically. However, authors of other reports emphasized the need for careful monitoring during skin-to-skin contact. Bohnhorst et al. reported an increase of bradycardia and hypoxia during skin-to-skin care (6). In contrast, in low-birth weight infants kangaroo care with skin-to-skin contact was shown to improve physiological parameters (7). Cases have been published describing need for cardiopulmonary resuscitation in the delivery room during skin-to-skin contact, in some cases leading to death (8–13). Skin-to-skin contact was also proven to be a risk factor for neonatal apparent life-threatening events or unexpected death in a prospective study with over 62,000 live births (14). The authors of these reports emphasize the need for careful observation during skin-to-skin contact. Therefore, monitoring of SpO_2_ seems useful during FCS procedures.

Continuous monitoring with pulse oximetry during the entire FCS procedure was performed in our study group. Also, since October 2013 pulse oximetry screening was performed at the maternity ward of the LUMC (15). Since there is more awareness for hypoxia and bradycardia, more cases of mild respiratory morbidity could have been reported. As a consequence, the admittance rate might have been increased in comparison to the standard CS when there was pulse oximetry monitoring only if judged necessary during evaluation at the resuscitation table. The additional monitoring with pulse oximetry might have led to overtreatment. However, we believe that hypoxia in newborns should always be monitored to assess if it is self-limiting or if there might be another underlying cause.

Infants born after FCS had normal median Apgar scores. The Apgar scores were statistically significant different among the groups, but this difference is not clinical significant and can be explained by some low outliers in the standard CS group in infants needing respiratory support.

One-third of the infants born after FCS were separated from their mother during or after the procedure, despite the goal of FCS to improve bonding. This suggests that the procedure of FCS should be evaluated carefully among all involved specialties and improvements in the protocol should be sought. For instance, the infant could be taken to the resuscitation table for initial assessment and stimulation by the pediatric resident, as is done in several other hospitals (16). By doing this, the neonatal transition might be enhanced, leading to better oxygenation, possibly reducing the need for monitor observation and admission. This short assessment and stimulation would only take little time, so skin-to-skin contact can be initiated shortly after birth, within the World Health Organization recommendation of 30 min, and bonding and initiation of breastfeeding will not be impaired (17).

It is known that there is an increased risk for respiratory morbidity in infants after elective CS when compared to infants born vaginally or after emergency CS (18, 19). A possible explanation is the lower catecholamine level when CS is performed before labor due to less stress of the infant, leading to less clearance of lung fluid. Increasing the GA to 39 weeks as a minimum for FCS should therefore be considered, and stricter adherence to the inclusion criterion of ≥38 weeks should at least be accomplished.

After FCS, the infants are laid down on the chest of mother. However, due to the sterile curtain for the surgical procedure, the space at the maternal chest is limited. The prone position of the infant during skin-to-skin contact with flexion of the neck can lead to oronasal obstruction and an increase in upper airway resistance. This can delay the clearance of lung fluid. The position of the infant and the posture of the neck should therefore be evaluated during skin-to-skin contact by the attending obstetric nurse.

There was a high rate of hypothermia in both groups. Despite the increase of temperature in the operation room, the use of a forced-air warming system and direct skin-to-skin contact, the rate of hypothermia in infants born after FCS was still 13%. These results have led to further measures to decrease the rate of hypothermia, including the use of warm blankets to cover the baby and mother during the procedure and in the post-operative recovery room.

The comparison of the FCS with a historical cohort is a limitation of the study. However, no other changes in protocols for planned CS occurred. Furthermore, the mothers with risk factors for neonatal morbidity were excluded in the standard CS group to make the groups more comparable.

This is an evaluation of practice at our hospital. Since this study was not randomized but compared two retrospective cohorts, an explanation of the higher admittance rate is difficult to determine. In our cohort, low SpO_2_ and hypoglycemia accounted for more than half (10/19) of all unplanned admissions. In order to assess the neonatal safety more comprehensively, a large prospective study is currently performed.

In conclusion, there was a higher admittance incidence of newborns after implementing FCS, without an increase in respiratory morbidity or hypothermia, so there are currently no concerns for newborn safety of the procedure. Low SpO_2_, which was preferably monitored in the FCS group, and hypoglycemia was responsible for a large part of the unplanned admissions. The LUMC will continue to perform FCS. However, measures to reduce separation of newborns from their mother after FCS should be taken.

## Ethics Statement

All procedures performed in this study were in accordance with the ethical standards of the institutional and national research committee and with the 1964 Helsinki declaration and its later amendments. Informed consent was not needed for this retrospective study with anonymized data obtained from medical charts based on standard care.

## Author Contributions

IN was the executive researcher of this study. She performed literature search, data collection, data analysis, data interpretation, writing, and submitting of the report. EM was involved in study design and critical revision of the content of the report. KB, JV, and LF were involved in data interpretation and editing of the report. VW was involved in literature search. AP was the project leader and performed literature search, designed the study, and coordinated data analysis, data interpretation, writing and editing of the report. All the authors gave approval for the final version to be published and agree to be accountable for all aspects of the work in ensuring that questions related to the accuracy or integrity of a part of the work are appropriately investigated and resolved. No honorarium, grant, or other form of payment was given to anyone to produce the manuscript.

## Conflict of Interest Statement

The authors declare that the research was conducted in the absence of any commercial or financial relationships that could be construed as a potential conflict of interest.

## Abbreviations

CS, caesarean section(s); FCS, family-centered caesarean section(s); HR, heart rate; LUMC, Leiden University Medical Center; NICU, Neonatal Intensive Care Unit; SpO_2_, peripheral oxygen saturation.
